# Zinc finger protein 382 is downregulated by promoter hypermethylation in pediatric acute myeloid leukemia patients

**DOI:** 10.3892/ijmm.2014.1966

**Published:** 2014-10-13

**Authors:** YAN-FANG TAO, SHAO-YAN HU, JUN LU, LAN CAO, WEN-LI ZHAO, PEI-FANG XIAO, LI-XIAO XU, ZHI-HENG LI, NA-NA WANG, XIAO-JUAN DU, LI-CHAO SUN, HE ZHAO, FANG FANG, GUANG-HAO SU, YAN-HONG LI, YI-PING LI, YUN-YUN XU, JIAN NI, JIAN WANG, XING FENG, JIAN PAN

**Affiliations:** 1 Department of Hematology and Oncology, Children’s Hospital of Soochow University, Suzhou, Jiangsu, P.R. China; 2 Department of Gastroenterology, The 5th Hospital of Chinese PLA, Yinchuan, Ningxia, P.R. China; 3 Department of Cell and Molecular Biology, Cancer Institute (Hospital), Chinese Academy of Medical Sciences, Peking Union Medical College, Beijing, P.R. China; 4 Translational Research Center, Second Hospital, The Second Clinical School, Nanjing Medical University, Nanjing, Jiangsu, P.R. China

**Keywords:** zinc finger protein 382, pediatric acute myeloid leukemia, methylation, tumor suppressor

## Abstract

Acute myeloid leukemia (AML) is the second-most common form of leukemia in children. Aberrant DNA methylation patterns are characteristic of AML. Zinc finger protein 382 (ZNF382) has been suggested to be a tumor suppressor gene possibly regulated by promoter hypermethylation in various types of human cancer. However, ZNF382 expression and methylation status in pediatric AML is unknown. In the present study, ZNF382 transcription levels were evaluated by quantitative reverse-transcription PCR. Methylation status was investigated by methylation-specific (MSP) PCR and bisulfate genomic sequencing (BGS). The prognostic significance of ZNF382 expression and promoter methylation was assessed in 105 cases of pediatric AML. The array data suggested that the ZNF382 promoter was hypermethylated in the AML cases examined. MSP PCR and BGS analysis revealed that ZNF382 was hypermethylated in leukemia cell lines. Furthermore, treatment with 5-aza-2′-deoxycytidine (5-Aza) upregulated ZNF382 expression in the selected leukemia cell lines. The aberrant methylation of ZNF382 was observed in 10% (2/20) of the control samples compared with 26.7% (28/105) of the AML samples. ZNF382 expression was significantly decreased in the 105 AML patients compared with the controls. Patients with ZNF382 methylation showed lower ZNF382 transcript levels compared with patients exhibiting no methylation. There were no significant differences in clinical characteristics or cytogenetic analysis between the patients with or without ZNF382 methylation. ZNF382 methylation correlated with minimal residual disease (MRD). Kaplan-Meier survival analysis revealed similar survival times in the samples with ZNF382 methylation, and multivariate analysis revealed that ZNF382 methylation was not an independent prognostic factor in pediatric AML. The epigenetic inactivation of ZNF382 by promoter hypermethylation can be observed in AML cell lines and pediatric AML samples. Therefore, our study suggests that ZNF382 may be considered a putative tumor suppressor gene in pediatric AML. However, further studies focusing on the mechanisms responsible for ZNF382 downregulation in pediatric leukemia are required.

## Introduction

Acute myeloid leukemia (AML) is defined as a clonal disorder caused by the malignant transformation of a bone marrow-derived, self-renewing stem cell or progenitor, which demonstrates a decreased rate of self-destruction, as well as aberrant differentiation (1). AML consists of a group of malignant disorders which are characterized by the replacement of normal bone marrow with abnormal, primitive hematopoietic cells (2). If left untreated, the disorder uniformly results in death, usually due to infection or bleeding. Pediatric AML comprises up to 20% of all childhood leukemia cases. Epigenetic disturbances have been implicated in the development and pathogenesis of leukemia (3). The inactivation of tumor suppressor genes by promoter hypermethylation has been increasingly recognized as a key event in leukemia, with the silencing of tumor suppressor genes by aberrant DNA hypermethylation reported in hematologic malignancies, including subsets of AML (4–6). Previous studies have identified DNA methylation of other tumor suppressor genes in AML, including retinoic acid receptor *β* (*RARβ*), estrogen receptor (*ER*)*,* p15 (7), *E-cadherin*, p16 (7), glutathione S-transferase Pi 1 (*GSTP1*) (8), hypermethylated in cancer 1 (*HIC1*) (9) and death-associated protein kinase 1 (*DAPK1*) (10). *DAPK1* is the most frequently methylated gene in both pediatric (70%) and adult AML (55%). *RARβ* is aberrantly methylated in 18% of AML cases and *p73* (11) is methylated in 10% of AML cases (8). Identifying novel aberrantly methylated genes may provide a better understanding of AML, thereby paving the way for the development of novel tumor markers and therapeutic targets (7,12,13).

Recently, epigenetic disturbances, such as aberrant promoter hypermethylation and covalent histone modifications (14), have been implicated in the pathogenesis of leukemia (3,15). In addition, these aberrations are responsible for enhanced proliferation and self renewal, differentiation arrest, as well as the impaired apoptosis of leukemic blasts (16). There are two major mechanisms of DNA methylation-associated gene silencing. The first mechanism is that methylation directly blocks activating transcription factors from binding to their target sequences. A number of transcription factors [activating protein 2 (AP-2), cAMP response element binding protein (CREB)/activating transcription factor (ATF), c-MYC and E2F family members] are known to bind unmethylated promoter sequences, but fail to bind at methylated sites. The second mechanism involves the acquisition of methyl-binding domain proteins [methyl-CpG-binding domain protein 1 (MBD1-4), methyl CpG binding protein 2 (Rett syndrome) (MeCP2) and Kaiso] and the recruitment of additional factors with repressive properties, such as histone deacetylases (17,18). The epigenetic control of gene expression can alter gene function without modification of the DNA coding sequence and has been suggested to play a fundamental role in normal and tumor cells (19).

Over the past few years, the role of genetic events in AML has been studied extensively, and a number of molecular defects and their contribution to leukemogenesis have been unraveled (20). However, epigenetic alterations in AML are not yet fully understood. Zinc finger (ZNF) proteins are among the most abundant proteins in eukaryotic genomes. ZNF protein functions are extraordinarily diverse and include DNA recognition, RNA packaging, transcriptional activation, regulation of apoptosis, protein folding and assembly, as well as lipid binding. ZNF proteins play important roles in a variety of physiological processes. ZNF transcription factors are involved broadly in development and tumorigenesis (21,23,29). The knockdown and overexpression of ZNF280B protein have demonstrated that ZNF280B is involved in both pro-growth and pro-survival functions in prostate cancer. However, surprisingly, the pro-cancer functions of ZNF280B are mediated by the downregulation of p53 (21). ZNF300 expression enhances nuclear factor (NF)-κB signaling by activating TNF receptor-associated factor 2 (TRAF2) and physically interacting with IKKβ. Furthermore, ZNF300 overexpression increases extracellular signal-regulated kinase (ERK)1/2 phosphorylation. It has been demonstrated that ZNF300 overexpression stimulates prostate cancer cell proliferation *in vitro* and significantly enhances tumor development and metastasis in a mouse xenograft model (22). The overexpression of ZNF425 inhibits the transcriptional activities of serum response element (SRE), AP-1 and serum response factor (SRF); deletion analysis indicates that the C2H2 domain is the main region responsible for the repression. The ZNF425 gene is a novel transcriptional inhibitor that functions in the MAPK signaling pathway (23).

ZNF382 functions as a tumor suppressor in multiple carcinomas. It has been shown that the ectopic expression of ZNF382 in silenced tumor cells significantly inhibits their clonogenicity and proliferation, and induces apoptosis (24). ZNF382 has been shown to be frequently methylated in multiple primary tumors, such as nasopharyngeal, esophageal, colon, gastric and breast cancer (24). To the best of our knowledge, there are no studies available to date on the expression of ZNF382 and the methylation status of its promoter in pediatric AML. In this study, we provide the first evidence of ZNF382 methylation in both AML cell lines and pediatric myeloid leukemia samples. These results suggest that ZNF382 functions as a tumor suppressor in pediatric AML.

## Materials and methods

### Cell lines

The leukemia cell lines, HL-60, MV4-11, U937 and K562, were obtained from the American Type Culture Collection (ATCC, Manassas, VA, USA). The Raji, Jurkat, 697, NB4, THP-1, Daudi and SHI-1 cell lines were kind gifts from Professor Wang Jian-Rong (Cyrus Tang Hematology Center of Soochow University, Suzhou, China). All the cell lines were maintained at 37°C in RPMI-1640 medium (GibcoR; Life Technologies, Carlsbad, CA, USA) supplemented with 10% fetal bovine serum (Invitrogen, Life Technologies, Carlsbad, CA, USA).

### Patients and samples

Bone marrow specimens were obtained at the time of diagnosis during the routine clinical assessment of 105 pediatric patients with AML, who presented at the Department of Hematology and Oncology, Children’s Hospital of Soochow University between 2006 and 2011. Ethical approval was provided by the Children’s Hospital of Soochow University Ethics Committee (nos. SUEC2006-011 and SUEC2000-021), and informed consent was obtained from the parents or guardians. AML diagnosis was made in accordance with the revised French-American-British (FAB) classification. ZNF382 expression and methylation were assessed in 105 Chinese pediatric AML patients with clinical follow-up records, including patient age, gender, FAB, cytogenetic and survival information. Multivariate analysis of ZNF382 expression or promoter methylation was also performed. Additionally, bone marrow samples from 12 healthy donors and 8 patients with idiopathic thrombocytopenic purpura (ITP) were analyzed as the controls. Bone marrow mononuclear cells (BMNCs) were isolated using Ficoll solution within 2 h after the bone marrow samples harvested. Analysis of the methylation status of genes in the pediatric AML samples (M1, M2, M3, M4 and M5) and 3 NBM control samples (N1, N2 and N3) was carried out using the NimbleGen Human DNA Methylation 385K Promoter Plus CpG Island Array. The microarray analysis was performed by KangChen Bio-tech, Shanghai P.R. China.

### CD34^+^ cell purification

For the selection CD34^+^ cells, the Miltenyi immunoaffinity device (VarioMACS 130-046-703) was used according to the instructions provided by the manufacturer. (Miltenyi Biotech, Auburn, CA, USA). Briefly, the CD34^+^ cells were magnetically labeled with CD34 MicroBeads. Subsequently, the cell suspension was loaded onto a MACSR Column which was placed in the magnetic field of a MACS Separator. The magnetically labeled CD34^+^ cells were retained within the column. The unlabeled cells ran through; the CD34^+^ cells were adsorbed on the magnetic poles. Following the removal of the column from the magnetic field, the magnetically retained CD34^+^ cells were eluted as the positively selected cell fraction.

### Sodium bisulphite modification of genomic DNA

High-molecular-weight genomic DNA was extracted from the cell lines and biopsy samples by a conventional phenol/chloroform method. The sodium bisulphite modification procedure was carried out as previously described with slight modifications (25). In brief, 600 ng of genomic DNA were denatured in 3 M NaOH for 15 min at 37°C, then mixed with 2 vol 2% low-melting-point agarose. Agarose/DNA mixtures were then pipetted into chilled mineral oil to form agarose beads. Aliquots of 200 μl of 5 M bisulphite solution (2.5 M sodium metabisulphite and 100 mM hydroquinone, both from Sigma-Aldrich, St. Louis, MO, USA) were added to each tube containing a single bead. The bisulphite reaction was then carried out by incubating the reaction mixture for 4 h at 50°C in the dark. Treatments were terminated by equilibration against 1 ml of TE buffer, followed by desulphonation in 500 μl of 0.2 M NaOH. Finally, the beads were washed with 1 ml of TE buffer and used directly for PCR.

### Methylation-specific (MSP) PCR

The methylation status of the ZNF382 promoter region was determined by MSP PCR analysis. Primers distinguishing unmethylated (U) and methylated (M) alleles were designed with MethPrimer (http://www.urogene.org/methprimer/) to amplify the sequence: ZNF382 B M forward, 5′-AGGTTAGGGAGGTAATTAA GAGTTC-3′ and reverse, 5′-GAATCACCCTCAAAATC ACG-3′; ZNF382 B U forward, 5′-GAGGTTAGGGAGGTAA TTAAGAGTTT-3′ and reverse, 5′-CAAATCACCCTCAAAA TCACAC-3′.

Each PCR reaction contained 20 ng of sodium bisulphite-modified DNA, 250 pmol of each primer, 250 pmol deoxynucleoside triphosphate, 1× PCR buffer, and one unit of ExTaq HS polymerase (Takara, Tokyo, Japan) in a final reaction volume of 20 μl. Cycling conditions were initial denaturation at 95°C for 3 min, 40 cycles of 94°C for 30 sec, 65 (M) or 63°C (U) for 30 sec and 72°C for 30 sec. For each set of methylation-specific PCR reactions, *in vitro*-methylated genomic DNA treated with sodium bisulphite served as a positive methylation control. PCR products were separated on 4% agarose gels, stained with ethidium bromide and visualized under UV illumination. For cases with borderline results, PCR analyses were repeated.

### Bisulfite genomic sequencing (BGS)

BGS was performed as previously described (24). Primers were designed with MethPrimer (http://www.urogene.org/methprimer/). The primers for BGS analysis were from +132 to +431, including 37 CpGs: ZNF382 forward, 5′-AGGTTAGGGAGGTAATTAAGAGTT-3′ and reverse, 5′-AACACCTAAAATACAACAAATTAAAC-3′. Amplified BGS products were TA-cloned; and 5 to 6 randomly selected colonies were sequenced. DNA sequences were analyzed with QUMA analyzer. (http://quma.cdb.riken.jp/).

### Leukemia cells treated with 5-aza-2′-deoxycytidine (5-Aza-dC)

Demethylation was induced with 5-Aza-dC (5-Aza; Sigma-Aldrich) treatment at a concentration that induced demethylation of the DNA without killing the cells. The culture medium for the HL-60 and MV4-11 cells contained 5 μM 5-Aza. DNA and RNA were extracted after 72 h of 5-Aza treatment for the following analysis. A group treated with DMSO was used as the control group.

### Quantitative reverse-transcription PCR (RT-qPCR) for ZNF382

RT-qPCR was performed to determine the expression levels of the ZNF382 gene. Total RNA was reverse transcribed using the Reverse Transcription kit, according to the manufacturer’s instructions (Applied Biosystems Inc., Foster City, CA, USA). The real-time PCR primers used to quantify GAPDH expression were: forward, 5′-AGAAGGCTGGGGCTCATTTG-3′ and reverse, 5′-AGGGGCCATCCACAGTCTTC-3′; and for ZNF382 forward, 5′-TGAGGGGTCTAGAATGCCTG-3′ and reverse, 5′-TGGACACAGAGAATTTTTCCA-3′. Real-time PCR was performed using FastStart SYBR-Green Master (cat. no. 04673484001) on a LightCycler480 system [both from Roche Diagnostics (Schweiz) AG, Rotkreuz, Switzerland]. The expression of ZNF382 was normalized to endogenous GAPDH expression.

### Statistical analysis

SPSS software version 11.5 (SPSS Inc., Chicago, IL, USA) was used for statistical analysis. Data are presented as the means ± standard deviation. A group t-test was used to compare the expression of ZNF382 between the DMSO group and the 5-Aza group. Statistical significance between the methylated sample data and clinicopathological characteristics of the AML patients were analyzed by Pearson’s Chi-square test or Fisher’s exact test. Statistical significance of ZNF382 expression among normal bone marrow (NBM) and pediatric AML groups was determined using one-way ANOVA. A value of P<0.05 was considered to indicate a statistically significant difference.

## Results

### ZNF382 promoter is hypermethylated in AML cells

**Figure 1:**
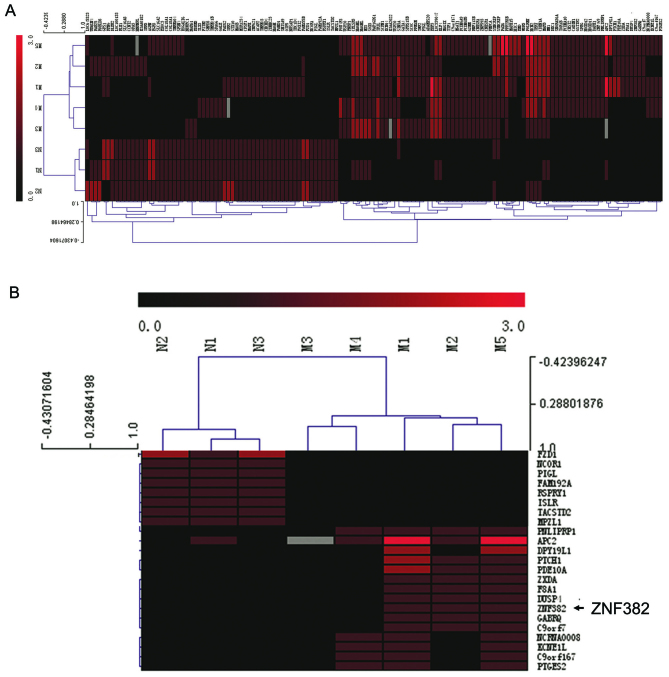
Promoter methylation analysis of pediatric acute myeloid leukemia (AML) samples. (A) Analysis of the methylation status of genes in pediatric AML samples (M1, M2, M3, M4 and M5) and 3 NBM control samples (N1, N2 and N3) using NimbleGen Human DNA Methylation Arrays. Each red box represents the number of methylation peaks (PeakScore) overlapping the promoter region for the corresponding miRNA. The PeakScore is defined as the average -log10 (P-value) from probes within the peak. The scores reflect the probability of positive methylation enrichment. (B) DNA methylation array analysis shows significant promoter zinc finger protein 382 (ZNF382) methylation in AML samples and no ZNF382 methylation in NBM control samples.

**Figure 2:**
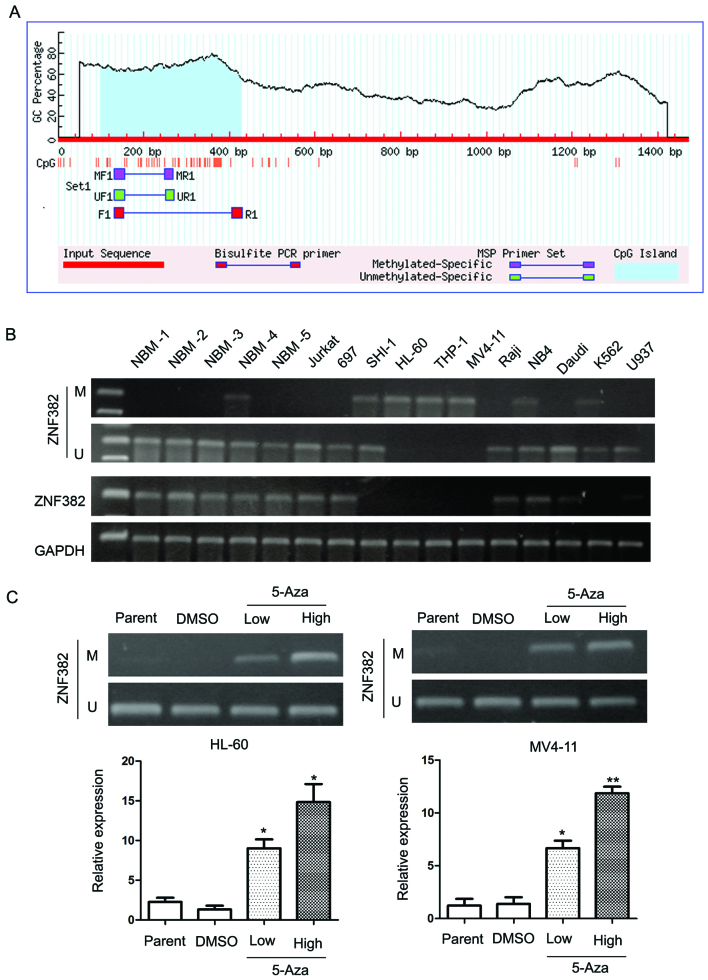
The zinc finger protein 382 (ZNF382) promoter is methylated in acute myeloid leukemia (AML) cell lines. (A) CpG island regions can be identified in the promoter of ZNF382. (B) Methylation analysis by methylation-specific PCR (MSP) shows ZNF382 hypermethylation in AML cell lines. M and U represent MSP results using primer sets for methylated and unmethylated ZNF382 genes, respectively. (C) The ZNF382 transcript level is upregulated in cells treated with 5-Aza compared with the DMSO control. Parent indicates the parent cell line.

ZNF382 is a functional tumor suppressor frequently methylated in multiple carcinomas, including nasopharyngeal, esophageal, colon, gastric and breast cancer (24). However, the methylation status of ZNF382 in the blood system is unknown, particularly in pediatric AML. Our analyses of promoter methylation in pediatric AML suggested that the ZNF382 promoter was hypermethylated in AML using NimbleGen Human DNA Methylation 385K Promoter plus CpG Island Arrays (Fig. 1A). We found that the ZNF382 promoter was hypermethylated in 3/5 (60%) pediatric AML samples and in 0/3 (0%) normal bone marrow samples (Fig. 1B). Subsequent analyses of the ZNF382 promoter sequence identified a large CpG island (Fig. 2A). MSP PCR assays were performed to detect the methylation status of the ZNF382 promoter in the 11 leukemia cell lines. The MSP primer was designed using MethPrimer (http://www.urogene.org/cgi-bin/methprimer/methprimer.cgi) and encompassed the CpG islands of the ZNF382 promoter identified in Fig. 2A. Our results revealed that the ZNF382 promoter was hypermethylated in 6 leukemia cell lines, including SHI-1, HL-60, THP-1, MV4-11, NB4 and K562 cells (Fig. 2B). In addition, ZNF382 expression was detected in 5 cell lines (Jurkat, 697, Raji, NB4 and Daudi) by RT-qPCR (Fig. 2B). These results suggest that the downregulation of ZNF382 in AML cells is a common phenomenon. To confirm the methylation of the ZNF382 promoter, we treated the leukemia cell lines with the demethylation reagent, 5-Aza. 5-Aza is an epigenetic modifier that inhibits DNA methyltransferase activity through the remodeling (opening) of chromatin, which results in DNA demethylation (hypomethylation) and gene activation. Our results revealed that treatment with 5-Aza significantly upregulated ZNF382 expression. As shown in Fig. 2C, ZNF382 expression was upregulated by 11.1-fold in the HL-60 cells (5-Aza 14.83 vs. DMSO 1.33; P=0.023) and by 8.47-fold in the MV4-11 cells (5-Aza 11.87 vs. DMSO 1.4; P<0.001). Furthermore, these results were supported by MSP analyses, which revealed a change in the methylation status of the ZNF382 promoter following treatment with 5-Aza. In summary, these results demonstrate that the ZNF382 promoter is consistently methylated in human myeloid leukemia cell lines. Based on these findings, we suggest that the ZNF382 promoter may be methylated in pediatric AML patients.

### The ZNF382 promoter is methylated in pediatric AML patients

**Figure 3:**
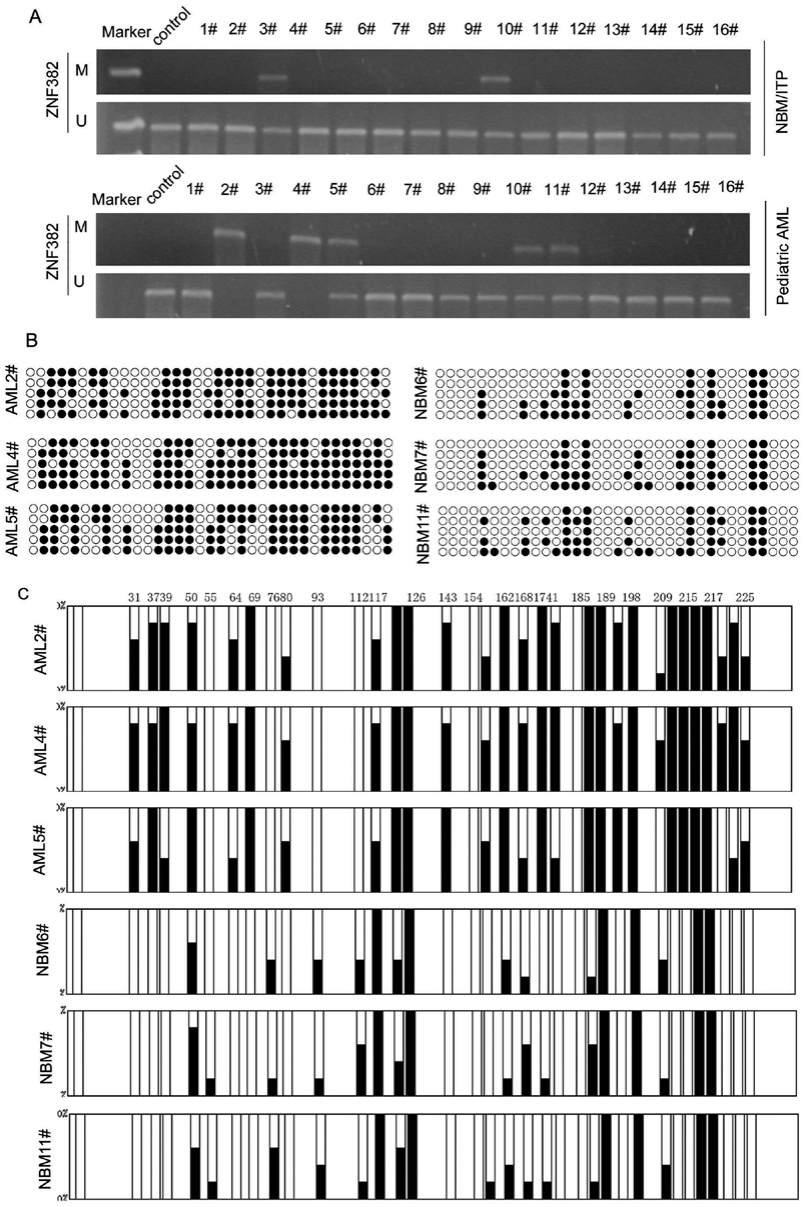
Zinc finger protein 382 (ZNF382) is inactivated by promoter hypermethylation in pediatric acute myeloid leukemia (AML). (A) Methylation-specific PCR (MSP) analysis of the methylation status of ZNF382 shows aberrant methylation in pediatric AML samples compared with normal bone marrow/idiopathic thrombocytopenic purpura (NBM/ITP) control samples. M and U represent MSP results using primer sets for methylated and unmethylated ZNF382 genes, respectively. (B) A total of 3 NBM samples and 3 AML samples were analyzed by bisulfite genomic sequencing (BSG), ●, methylated cytosines; ○, unmethylated cytosines. (C) The results revealed that the CpG islands in the ZNF382 promoter were methylated in the AML samples (63.4, 70.9 and 60.0% in AML2#, AML4# and AML5#, respectively). By contrast, the CpG islands of ZNF382 promoter in the NBM samples were unmethylated (26.3, 29.1 and 29.1% in NBM6#, NBM7# and NBM11#, respectively).

We then examined the methylation status of the ZNF382 promoter in pediatric AML samples and normal bone marrow (NBM)/idiopathic thrombocytopenic purpura (ITP) control samples. The aberrant methylation of ZNF382 was observed in 26.7% (28/105) of the pediatric AML samples compared with 10% (2/20) of the control samples (Fig. 3A). A total of 3 control samples and 3 AML samples were further analyzed by BGS (Fig. 3B). The BGS results revealed that CpG islands in the ZNF382 promoter were methylated in the AML samples (63.4, 70.9 and 60% in AML2#, AML4# and AML5#, respectively). By contrast, CpG islands in the ZNF382 promoter were unmethylated in the control samples (26.3, 29.1 and 29.1% in NBM6#, NBM7# and NBM11#, respectively). Furthermore, these results were supported by MSP assays.

### ZNF382 expression is downregulated by promoter methylation in pediatric AML patients

**Figure 4:**
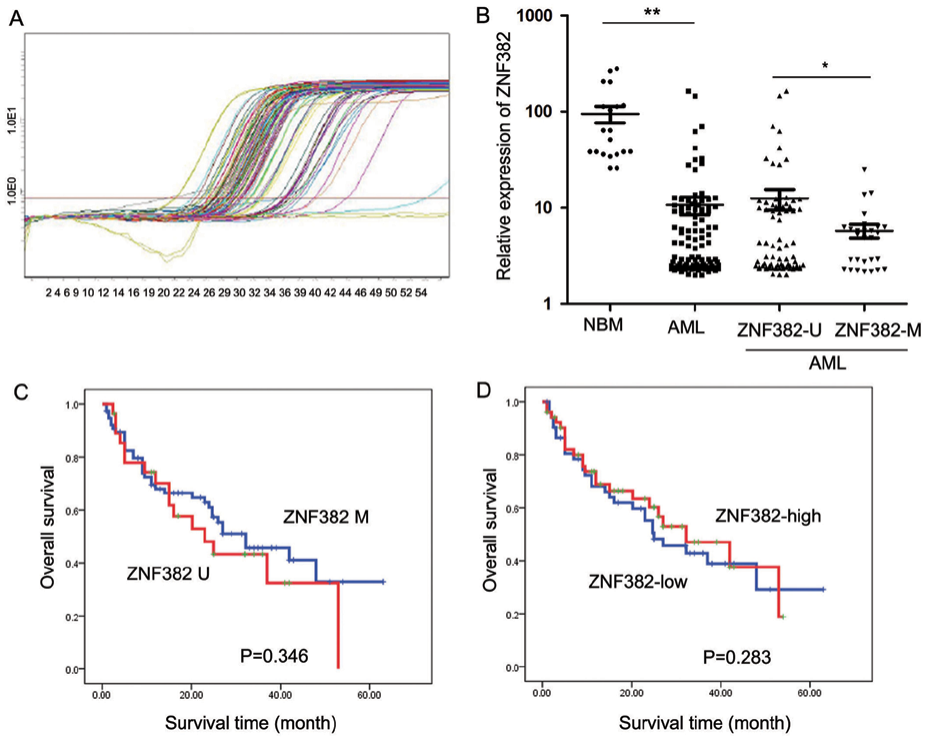
Expression of zinc finger protein 382 (ZNF382) is downregulated by promoter methylation in pediatric acute myeloid leukemia (AML). (A) The transcript levels of ZNF382 were examined in pediatric AML patients by RT-qPCR. (B) ZNF382 expression was significantly decreased in the 105 AML patients (10.73±23.01; P<0.001) compared to the 20 normal bone marrow/idiopathic thrombocytopenic purpura (NBM/ITP) controls (94.74±81.62); patients with ZNF382 methylation (5.77±4.90, n=28) showed lower ZNF382 transcript levels compared to those without no ZNF382 methylation (12.53±26.53, P=0.043; n=77); and both those AML patients with and without ZNF382 methylation showed significantly lower ZNF382 transcript levels compared to the controls. ^*^P<0.05; ^**^P<0.01. (C) Kaplan-Meier survival analysis depicting ZNF382 promoter methylation in pediatric AML patients. Methylated ZNF382 is shown by the blue line, and unmethylated ZNF382 is shown by the red line. (D) Kaplan-Meier survival analysis depicting ZNF382 high (red line) or low (blue line) expression in pediatric AML patients.

The transcript levels of ZNF382 were examined in 105 pediatric AML patients by RT-qPCR (Fig. 4C). As shown in Fig. 4D, ZNF382 expression was significantly decreased in the 105 AML patients (10.73±23.01; P<0.001) compared with the 20 control samples (94.74±81.62). Patients exhibiting ZNF382 methylation (5.77±4.90, n=28) showed lower ZNF382 transcript levels compared to the patients without ZNF382 methylation (12.53±26.53, P=0.043; n=77). Furthermore, AML patients with and without ZNF382 methylation presented with significantly lower ZNF382 transcript levels compared with the controls (Fig. 4D). The prognostic significance of ZNF382 expression was assessed in 105 pediatric AML patients with clinical follow-up records. We found no significant association between ZNF382 expression and patient age, gender, FAB scores or cytogenetics (Table I). Kaplan-Meier survival analysis of the 105 pediatric AML patients revealed similar survival times in tumors with high or low ZNF382 expression (P=0.283) (Table III and Fig. 4B). Furthermore, multivariate analysis revealed that ZNF382 promoter methylation failed to be an independent prognostic factor in pediatric AML (P=0.832; Table IV).

### Promoter methylation of ZNF382 does not correlate with survival in pediatric AML

The prognostic significance of ZNF382 promoter methylation was assessed in 105 cases of pediatric AML patients with clinical follow-up records. Table II shows that ZNF382 promoter methylation correlated with minimal residual disease (MRD; P=0.025). We found no significant differences in clinical characteristics, such as gender, age, FAB scores or cytogenetics between the patients with or without ZNF382 methylation (Table II). Kaplan-Meier survival analysis revealed similar survival times in the samples with ZNF382 promoter methylation (P=0.346) (Table III and Fig. 4A). Furthermore, multivariate analysis revealed that ZNF382 promoter methylation failed to be an independent prognostic factor in pediatric AML (P=0.249) (Table IV).

In summary, our results demonstrated that the ZNF382 promoter was significantly methylated in leukemia cell lines. The aberrant methylation of ZNF382 was observed in 26.7% (28/105) of the pediatric AML samples compared with 10% (2/20) of the control samples. The expression of ZNF382 was significantly lower in the pediatric AML samples compared with the control samples, and patients with ZNF382 methylation had lower ZNF382 transcript levels compared to the patients with no ZNF382 methylation. Furthermore, ZNF382 promoter methylation correlated with MRD, and Kaplan-Meier survival analysis revealed similar survival times in samples harboring ZNF382 promoter methylation. Moreover, multivariate analysis revealed that ZNF382 promoter methylation failed to be an independent prognostic factor in pediatric AML.

## Discussion

Aberrant DNA methylation patterns are a characteristic feature of cancer, including myeloid malignancies, such as AML. In AML, the presence of common methylation patterns in a few genes, such as *p15* and *E-cadherin* has been independently described by several groups across larger patient cohorts (26,27). The progression from myelodysplastic syndrome to AML has been associated with increased aberrant DNA methylation. Genome-wide methylation patterns in AML have, however, thus far only been addressed in a few studies (4,28). In particular, to the best of our knowledge, whole-genome bisulfite sequencing analyses in AML are limited. DNA methylation coupled with the H3K9me3-mediated gene silencing of ZNF genes is widespread, occurring at individual ZNF genes on multiple chromosomes and across ZNF gene family clusters (29). In the present study, promoter methylation in pediatric AML was analyzed using NimbleGen Human DNA Methylation 385K Promoter plus CpG Island Arrays. This approach revealed significant differences in the methylation status of genes between pediatric AML and normal bone marrow samples. Our results suggested that the ZNF382 promoter was hypermethylated in AML. The ZNF382 promoter was hypermethylated in 3/5 (60%) of the pediatric AML samples and in 0/3 (0%) of the normal bone marrow samples.

ZNF382 is a functional tumor suppressor that is frequently methylated in multiple carcinomas, including nasopharyngeal, esophageal, colon, gastric and breast cancer (24). A genome-wide analysis of promoter associated CpG island methylation in acute lymphoblastic leukemia (ALL) has revealed DNA methylation of ZNF382 in primary ALL samples (30). To the best of our knowledge, this is the first study to determine the expression of ZNF382 and promoter methylation status in pediatric AML.

The molecular function of ZNF382 has been investigated in multiple carcinomas. As previously demonstrated, the ectopic expression of ZNF382 in silenced tumor cells significantly inhibits clonogenicity, proliferation and induces apoptosis. ZNF382 inhibits NF-κB and AP-1 signaling and downregulates the expression of multiple oncogenes, including *MYC,* microphthalmia-associated transcription factor *(MITF*)*,* high mobility group AT-hook 2 *(HMGA2*) and cyclin-dependent kinase 6 (*CDK6*), as well as the NF-κB upstream factors, including signal transducer and activator of transcription (STAT)3, STAT5B, inhibitor of DNA binding 1, dominant negative helix-loop-helix protein (ID1) and inhibitor of kappa light polypeptide gene enhancer in B-cells, kinase epsilon (IKBKE), most likely through heterochromatin silencing (24). ZNF family proteins play important roles in recognition for methylated CpG. Two recent structures of C2H2 ZNF proteins in complex with methylated DNA reveal a common recognition mode for 5-methylcytosine (5 mC) that involves a 5 mC-Arg-G triad. In the two ZNF proteins, an arginine that precedes the first Zn-binding histidine (RH motif) can interact with a 5 mCpG or TpG dinucleotide. RH-ZNF motifs provide specificity for 5 mCpG, whereas the neighboring ZNFs recognize the surrounding DNA sequence context (31). The ZNF protein, Kaiso, recognizes methylated and specific DNA sequences. Kaiso is a bifunctional Cys(2)His(2) ZNF protein implicated in tumor cell proliferation. Kaiso binds to both methylated CpG (mCpG) sites and specific unmethylated DNA motifs (TCCTGCNA) and represses transcription by recruiting chromatin remodeling corepression machinery to target genes (32). The ZNF proteins, zinc finger and BTB domain containing 4 (ZBTB4) and zinc finger and BTB domain containing 38 (ZBTB38), can also recognize methylated DNA. Kaiso, ZBTB4 and ZBTB38 can bind methylated DNA in a sequence-specific manner (33), suggesting that many other ZNF proteins, including ZNF382, may also have a similar function. Currently, the molecular function of ZNF382 in pediatric AML remains unknown and further investigations are required to elucidate the role of ZNF382 in pediatric leukemia. The epigenetic inactivation of ZNF382 by promoter hypermethylation can be observed in both AML cell lines and pediatric AML samples. Therefore, our study suggests that ZNF382 may be considered a putative tumor suppressor gene in pediatric AML. In addition, our findings indicate that ZNF382 promoter methylation correlates with MRD. Furthermore, Kaplan-Meier survival analysis revealed similar survival times in samples with ZNF382 promoter methylation. However, further studies focusing on the mechanisms responsible for ZNF382 methylation in pediatric leukemia are required.

## Figures and Tables

**Table I:** Association of ZNF382 expression with clinicopathological characteristics in the 105 pediatric AML samples.

Clinical variables	No. of patients	ZNF382 expression (n)		P-value

		Low	High	
Gender				
Male	42	20	22	0.633
Female	63	33	30	
Age (years)				
<6	60	34	26	0.143
≥6	45	19	26	
Leukocytes (/μl)				
>10,000	61	30	31	0.755
≤10,000	44	23	21	
FAB score				
M1-M6	93	45	48	0.233
M7	12	8	4	
Cytogenetics				
Favorable	50	25	25	0.639
Intermediate	27	12	15	
Unfavorable	28	16	12	
MRD				
<0.25%	49	24	25	0.774
≥0.25%	56	29	27	

ZNF382, zinc finger protein 382; AML, acute myeloid leukemia; FAB, French-American-British classification; MRD, minimal residual disease.

**Table II:** Association of ZNF382 promoter methylation with clinicopathological characteristics in the 105 pediatric AML samples.

Clinical variables	No. of patients	ZNF382 methylation (n)		P-value

		Negative	Positive	
Gender				
Male	42	30	12	0.719
Female	63	47	16	
Age (years)				
<6	60	42	18	0.372
≥6	45	35	10	
Leukocytes (/μl)				
>10,000	61	43	18	0.438
≤10,000	44	34	10	
FAB score				
M1-M6	93	71	22	0.052
M7	12	6	6	
Cytogenetics				
Favorable	50	36	14	0.955
Intermediate	27	20	7	
Unfavorable	28	21	7	
MRD				
<0.25%	49	41	8	0.025
≥0.25%	56	36	20	

ZNF382, zinc finger protein 382; AML, acute myeloid leukemia; FAB, French-American-British classification; MRD, minimal residual disease.

**Table III:** Association of ZNF382 expression and promoter methylation with Kaplan-Meier survival in the 105 pediatric AML samples.

Variable	No. of patients	Over survival Median ± SE	P-value
Cytogenetics			
Favorable	50	46.664±3.717	<0.001
Intermediate	27	29.220±3.188	
Unfavorable	28	11.161±1.827	
FAB score			
M1-M6	93	36.113±2.885	<0.001
M7	12	8.542±1.820	
Leukocytes (/μl)			
>10,000	61	30.220±2.974	0.803
≤10,000	44	33.631±4.063	
MRD			
<0.25%	49	53.627±3.151	<0.001
≥0.25%	56	18.893±2.425	
ZNF382 expression			
Low <4.03	52	32.020±3.661	0.283
High ≥4.03	53	31.571±3.306	
ZNF382 methylation			
Negative	77	34.215±3.236	0.346
Positive	28	28.051±4.275	

ZNF382, zinc finger protein 382; FAB, French-American-British classification; AML, acute myeloid leukemia; MRD, minimal residual disease.

**Table IV:** Cox multivariate analysis of ZNF382 expression, promoter methylation and clinicopathological characteristics in pediatric AML.

Variable	Odds ratio	EXP (B) 95% CI	P-value
Cytogenetics			
Fav. vs. inter and unfav.	4.569	2.216 (1.068–4.595)	0.033
MRD			
<0.25 vs. ≥0.25%	16.575	6.255 (2.588–15.119)	0.000
Leukocytes (/μl)			
>10,000 vs. ≤10,000	0.083	1.088 (0.615–1.925)	0.773
FAB score			
M7 vs. M1-M6	9.265	3.113 (1.498–6.469)	0.002
ZNF382 expression			
Low vs. high	0.045	0.939 (0.527–1.675)	0.832
ZNF382 methylation			
Negative vs. positive	1.326	0.690 (0.367–1.298)	0.249

ZNF382, zinc finger protein 382; AML, acute myeloid leukemia; Fav., favorable; inter, intermediate; unfav., unfavorable; FAB, French-American-British classification; MRD, minimal residual disease.
